# Power-Doppler Ultrasonic Imaging of Peripheral Perfusion in Diabetic Mice

**DOI:** 10.1109/TBME.2024.3373254

**Published:** 2024-07-18

**Authors:** Somaye Babaei, Lawrence W. Dobrucki, Michael F. Insana

**Affiliations:** Department of Bioengineering, University of Illinois at Urbana-Champaign, USA.; Department of Bioengineering, University of Illinois at Urbana-Champaign, USA, and also with the Beckman Institute for Advanced Science and Technology, USA.; Department of Bioengineering, University of Illinois at Urbana-Champaign, IL 61801 USA, and also with the Beckman Institute for Advanced Science and Technology, USA

**Keywords:** Clutter filtering, endothelial dysfunction, exercise conditioning, hindlimb ischemia, peripheral arterial disease, type-2 diabetes

## Abstract

**Objective:**

We explored the capabilities of power-Doppler ultrasonic (PD-US) imaging without contrast enhancement for monitoring changes in muscle perfusion over time.

**Methods:**

Ischemic recovery was observed in healthy and type II diabetic male and female mice with and without exercise. In separate studies, perfusion was measured during and after 5-min ischemic periods and during four-week recovery periods following irreversible femoral ligation. A goal was to assess how well PD-US estimates tracked the diabetic-related changes in endothelial function that influenced perfusion.

**Results:**

The average perfusion recovery time following femoral ligation increased 47% in diabetic males and 74% in diabetic females compared with non-diabetic mice. Flow-mediated dilation in conduit arteries and the reactive hyperemia index in resistive vessels each declined by one half in sedentary diabetic mice compared with sedentary non-diabetic mice. We found that exercise reduced the loss of endothelial function from diabetes in both sexes. The reproducibility of perfusion measurements was limited primarily by our ability to select the same region in muscle and to effectively filter tissue clutter.

**Conclusions/Significance:**

PD-US measurements can precisely follow site-specific changes in skeletal muscle perfusion related to diabetes over time, which fills the need for techniques capable of regularly monitoring atherosclerotic changes leading to ischemic vascular pathologies.

## I. Introduction

PERIPHERAL arterial disease (PAD) of the lower extremities is a consequence of slowly progressive atherosclerosis that occurs systemically across vascular scales, from large conduit arteries to smaller resistive vessels [1]. PAD is a significant threat to healthy aging because of its association with progression toward critical limb ischemia, coronary heart disease, and stroke [1], [2].

PAD is prevalent in about 20% of the elderly population, especially in individuals with a history of risk factors that include diabetes, hypertension, dyslipidemia, obesity, smoking, and a sedentary lifestyle [2]. These risk factors damage endothelial function that promotes atherosclerosis leading to vascular blockages. Patients often present clinically with leg pain caused by insufficient muscle perfusion. PAD is under-diagnosed and under-treated in the primary care setting [1], [3] in part because only 10–15% of PAD patients display classical symptoms [4]. To effectively support a large at-risk population, there is a need [5], [11] for effective low-risk imaging techniques designed to monitor site-specific changes in deep-muscle perfusion.

The initial clinical diagnosis of PAD is based on the ankle-brachial index (ABI) [12]. In practice, patients with ABI < 0.9 are considered positive for PAD and may undergo angiography to search for vascular stenoses and clots in conduit arteries [1]. Brachial artery flow-mediated dilation (FMD) studies [49] and reactive hyperemia index measurements via peripheral artery tonometry (RH-PAT) [10] are combined with a positive ABI diagnosis to further search for endothelial dysfunction, an important early indicator of atherogenic factors [13]. Severely ischemic patients are referred for surgical or endovascular procedures, while less severe cases benefit from therapies that target risk-factor mitigation and restoration of a healthy vascular endothelium [4], [5], [6].

Although PAD symptoms are a consequence of reduced perfusion, perfusion imaging is not currently part of routine clinical practice [14]. Several groups are pursuing non-contrast-enhanced perfusion imaging methods with standard ultrasound instruments [15], [16], [17]. Safe, effective, low-cost imaging methods enable frequent monitoring of peripheral perfusion changes within peripheral muscles to detect PAD progression and responses to treatment.

Building on recent work [18], [19], [20], [25], this report describes applications of power-Doppler ultrasonic (PD-US) imaging in diabetic mice. These methods employ an ischemic-hindlimb model where perfusion varies predictably with time [21], [22], [23], [24], [25]. Following right femoral ligation, changes in muscle perfusion are monitored via PD-US over a four-week recovery. PD-US methods were validated through comparisons with other imaging modalities [24], [25].

In this study, we demonstrate the capabilities of PD-US for detecting perfusion changes associated with diabetes including the assessment of endothelial function. The independence of endothelial responses in conduit and resistance vessels suggests there is scale-dependent information about the risk of atherogenesis advancing toward coronary artery disease [7], [8]. Changes in the function of conduit vessel endothelium measures the risks associated with advancing age and hypertension. However, changes in the endothelial function of resistive vessels, which PD-US indirectly measures [25], indicate risks from metabolic imbalances associated with high body-mass index and diabetes that lead to PAD [9].

The investigation focuses on diabetes-related changes to the maximum loss of perfusion following femoral ligation, the time to full perfusion recovery, and the resistive-vessel reactive hyperemia (RH) index. High sensitivity to disease-specific changes in mice demonstrates that PD-US imaging is capable of routinely monitoring peripheral perfusion in at-risk patients.

## II. Methods

### A. Animal Preparation

**Fig. 1.**
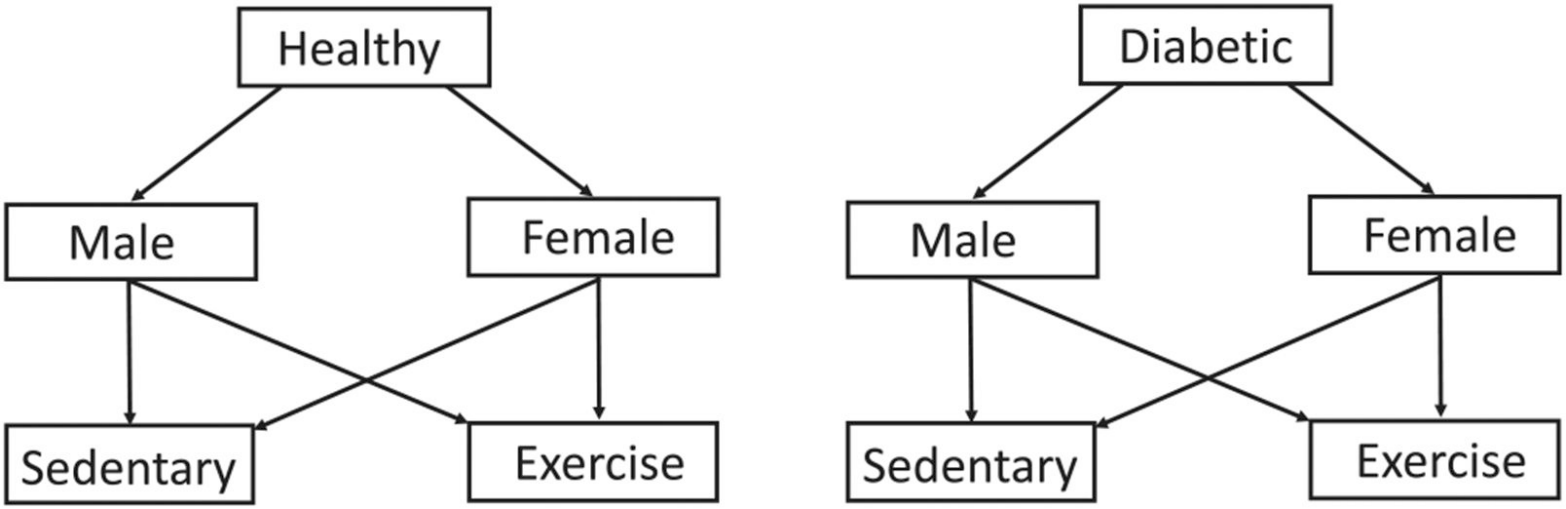
Breakdown of the mouse groups examined.

This study included 30 male and 30 female mice (C57BL/6, Charles River Laboratories, US). Eight subgroups are selected based on glucose-related health status, sex, and fitness level as outlined in Fig. 1. The Institutional Animal Care and Use Committee at the University of Illinois at Urbana-Champaign approved all procedures (Protocol number 20129).

Each mouse was anesthetized using isoflurane (2–3%) and placed in a supine position on a heating pad to maintain body temperature. Limbs are secured with Velcro straps, and the fur on the inner surface of both hindlimbs is removed using depilatory cream. The right femoral artery and vein are exposed through a small incision in the skin using micro scissors and gentle dissection. Two ligatures separated by 1 mm are placed on both vessels and cauterized between the ligatures to ensure a complete ligation. Ligating at the arterial site selected reduced downstream perfusion 30–50%. Flow patterns are naturally modified in collateral vessels to circumvent the loss and maintain enough perfusion to avoid muscle necrosis. Muscle perfusion returns to pre-ligation levels within two weeks as the limb undergoes normal arteriogenic and angiogenic compensatory processes [26], [27], [28].

**Fig. 2.**
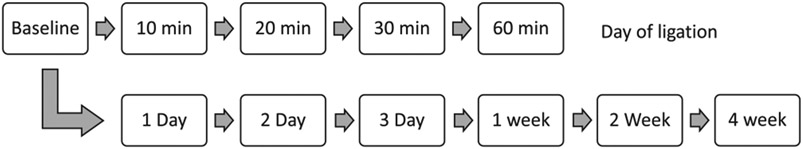
PD-US data are acquired from muscles in both mouse hindlimbs according to this measurement schedule. Baseline measurements are recorded before right femoral ligation at t = 0.

In addition to pre-ligation baseline measurements, PD-US data are acquired from muscles in the ischemic right hindlimb and the control left hindlimb at time points between 10 min and 4 weeks post-ligation, as indicated in the measurement schedule of Fig. 2.

### B. Diabetic Mouse Model

An obesity-induced type-2 diabetes mellitus mouse model was implemented for roughly half of the C57BL/6 mice [29], [30]. After a single low dose of streptozotocin (STZ, 20 mg/kg, i.p.) was injected to damage pancreatic *β* cells, mice were fed a high-fat, carbohydrate-free diet (HFD, Research Diets, cat #D12492, energy content: 72% lard, 28% protein, < 1% carbohydrate) for three months. The metabolic adaptation is sufficiently heterogeneous to expect only half of the treated mice to develop an obese diabetic phenotype [29]. Diabetic induction was confirmed with an intraperitoneal glucose tolerance assay. Briefly, glucose (1 g/kg) was injected i.p. in 6-hr fasted animals. Blood glucose levels were monitored in a 3.5 μL blood sample collected from a tail vein with a glucose meter at −30, 0, 30, 60, and 90 min after glucose administration [30]. A mouse was considered positive for diabetes when the fasting glucose baseline level (at T-30) was above 200 mg/dL, and both AUC(T0-T30) and AUC(T30-T90) reached statistically significant differences during glucose tolerance test versus non-diabetic animals [31].

The weight gain in this model is associated with insulin resistance and a lack of *β* cell compensation leading to impaired glucose tolerance. This model is considered an appropriate representation of human diabetic physiology because it is induced by environmental manipulation rather than genetic or chemical factors [32]. It is noteworthy that hyperglycemia induced by a single low-dose STZ administration is unlikely to cause diabetes in animals fed a standard diet [33].

### C. Exercise Regimen

As shown in Fig. 1, the numbers of healthy and diabetic male and female mice were each further divided into sedentary and exercised subgroups. Physical activity is an important factor determining vascular health in metabolic disorders [34]. Exercised mice underwent cardiovascular conditioning via forced swimming [35]. They initially swam for a few minutes each day, ramping up to 60 minutes per day over one week. Then, each mouse swam 60 minutes daily for two weeks immediately prior to ligation through four weeks of post-ligation testing.

### D. FMD and RH Estimates

We assessed endothelial function for the mouse groups by measuring flow-mediated dilation (FMD) of the left femoral artery and reactive hyperemia (RH) index via simultaneous PD-US perfusion estimates in muscles of the left (control) hindlimb. Our methods were described previously [25]. Briefly, following a 5-min pressure-cuff occlusion, we measured changes in the femoral artery diameter from 24 MHz B-mode ultrasound images every minute for 5 min. If *D*_*b*_ is the baseline vessel diameter before cuff inflation and *D*_*a*_ is the largest diameter during the hyperemic response approximately 1 min after pressure cuff release, FMD = (*D*_*a*_ − *D*_*b*_)/*D*_*b*_. We found that normal FMD values in mice ranged between 0.13 and 0.14 [25]. Values below this range indicate reduced endothelial function in conduit vessels.

From the same data acquisition, we measured PD-US in the downstream hindlimb muscle. Allowing PD-US_*b*_ to represent the baseline measurement before cuff inflation and PD-US*_a_* the estimate at the time of the peak hyperemic response, RH = 10 log(PD-US_*a*_/PD-US_*b*_). We found the range of normal response was 3.9 – 5.2 dB [25]. Smaller values indicate reduced endothelial function in resistance vessels.

### E. NO Inhibition

As perfusion declines following right femoral ligation, we observe a sudden short-lived perfusion peak 20 min after ligation. We hypothesize that the peak results from endothelium-dependent, flow-mediated nitric oxide (NO) release causing brief vasodilation as blood flow in the hindlimb adjusts to the sudden ischemia. To test the hypothesis, we conducted a separate study with 10 additional C57BL/6 mice. Five received an i.p. injection of non-specific nitric oxide synthase inhibitor L-NAME (Cayman Chemicals #80210, Ann Arbor, MI), 3 mg per mouse, one hour before right femoral ligation. Five additional mice that also underwent right femoral ligation served as controls. All 10 mice were healthy 9-month-old sedentary males.

### F. Power-Doppler Ultrasonic (PD-US) Measurements

Mice were prepared similarly to the surgical procedures, except anesthesia was reduced to 1.3% isoflurane and lasted only about 10 minutes per exam.

**Fig. 3.**
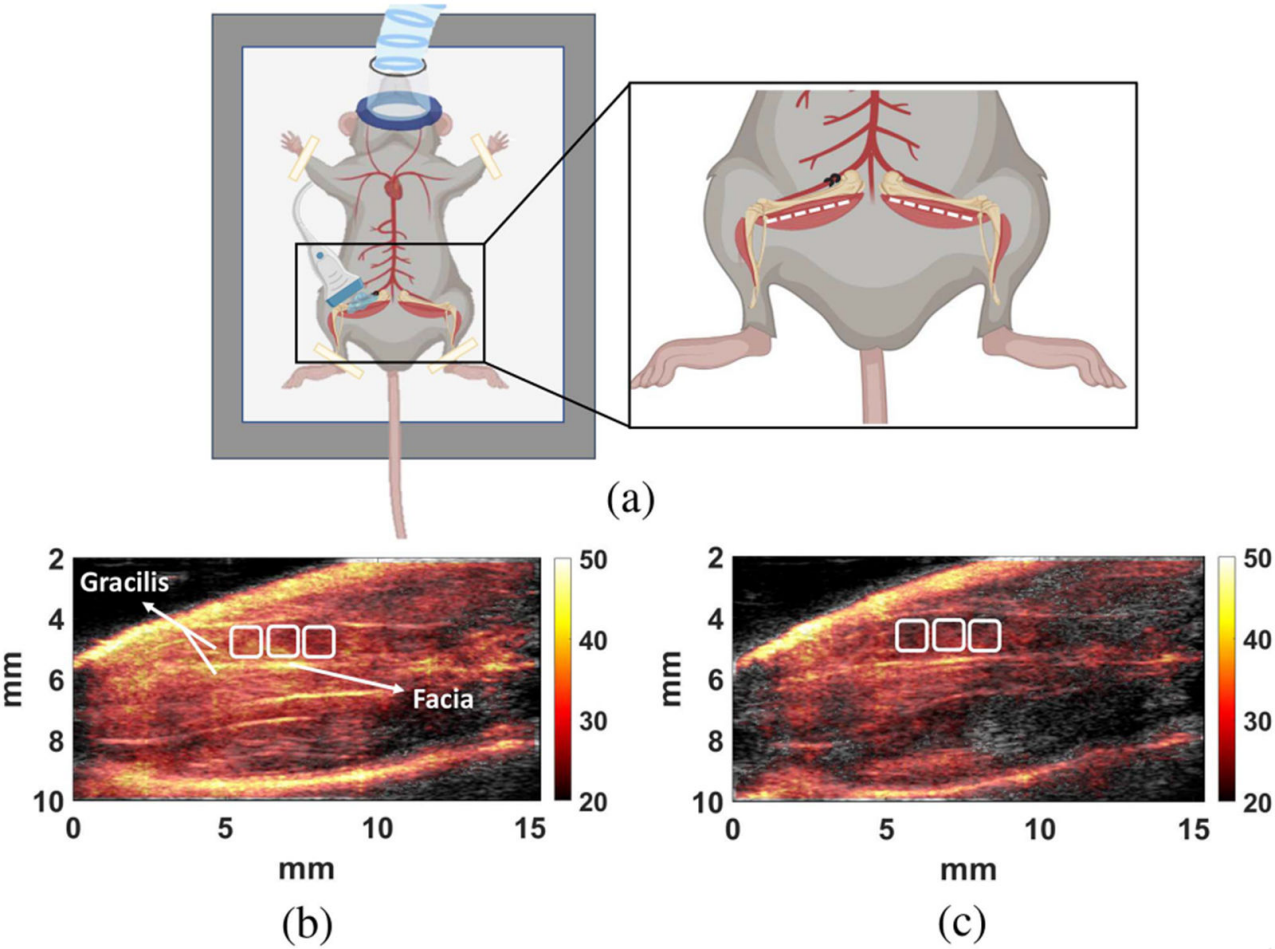
(a) Positioning of the 24 MHz linear array (not to scale) on a mouse wearing a nose cone to maintain anesthesia. Inset: the dashed white lines indicate the PD-US scan plane locations on muscles in both legs. (b) PD-US images of pre-ligation and 60-min post-ligation hindlimbs. Three 1 mm^2^ analysis regions in the gracilis muscle are selected to estimate the spatially averaged perfusion. Illustration in (a) was created with BioRender.com.

A Vevo 2100 system (FUJIFILM VisualSonics Inc., Toronto, Ontario, Canada) with an MS-400 probe was used to record pulse-echo signals. Scan planes for the right (ischemic) and left (control) hindlimb muscles are indicated as dashed lines in the inset of Fig. 3(a). Perfusion images are formed offline from radiofrequency (RF) echo data converted from the recorded in-phase and quadrature (IQ) data [55]. To locate the same image plane for each measurement, the probe is initially aligned to view the long axis of the femur. The probe is then translated medially and perpendicular to a scan plane until the femur is no longer visible. The probe is mechanically fixed at this location to eliminate hand jitter. Three 1 mm^2^ regions in the gracilis muscle are selected (see Fig. 3(b)).

The regions of interest for PD-US estimates are centered roughly 4 mm from the probe at a tissue depth of 2 mm, so that the ultrasonic attenuation losses are ~ 5 dB for all acquisitions ((0.5 dB)/(cm-MHz) × 2(0.2 cm) × 24 MHz). We do not compensate for attenuation because the signal loss is constant for all scans, giving an echo SNR of approximately 15 dB and resulting in acceptable PD-US estimation uncertainties [20]. Also, femoral ligation redistributes perfusion within the hindlimb as the body compensates for lost blood flow. Selecting the same muscle region at each time point minimizes the estimation variability caused by regional variations in perfusion as seen in Fig. 3.

**Fig. 4.**
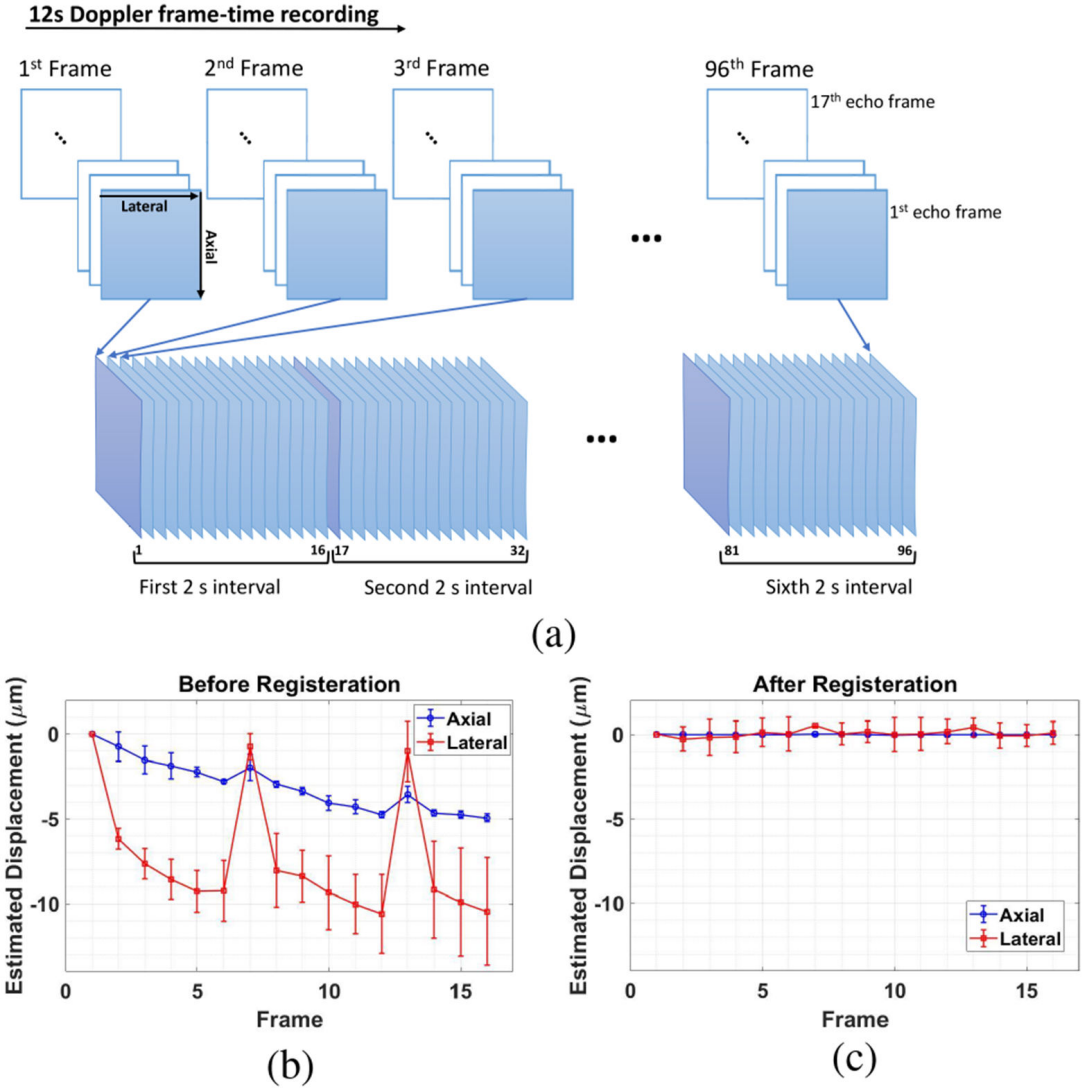
(a) Twelve seconds of echo data are recorded in 96 Doppler frames, each consisting of 17 color-mode echo frames. We select the first echo frame in each Doppler frame to assemble 2-s time intervals composed of 16 echo frames at 8 fps for each PD-US estimate. A 12-s recording yields PD-US estimates at six time points for one mouse; averaging values over *N* mice yields points plotted in Fig. 6. An example of relatively large echo displacements measured before (b) and after (c) applying rigid registration to the 16 frames in one 2 s interval is shown. Axial (blue) and lateral (red) movements are plotted as a function of time, where there are 0.125 s between frames.

The PD-US values reported are the result of averaging echo data over space, time, and the number of animals in a cohort. Ninety-six Doppler frames of echo data are recorded over 12 seconds at a rate of 8 fps (see Fig. 4(a)) and a transmit pulse frequency of 24 MHz. After each recording, at least 18 seconds are required to download the echo data from the scanner for offline processing before additional data can be recorded.

One Doppler frame consists of 17 echo frames acquired in color-flow mode at 1000 fps. We select the first echo frame from each of the 96 Doppler frames and partition the 96 echo frames into 6 groups of 16 echo frames each spanning 2 seconds of muscle perfusion. Hence signal power is estimated with 0.5 Hz Doppler-frequency resolution (0.016 mm/s velocity resolution [25]) and ±4 Hz bandwidth. A PD-US image was obtained to select three regions approximately 1 mm^2^ (Fig. 3(b)) for spatially averaged PD-US estimates at each time point. Given the speed of capillary flow in muscle (< 2 mm/s), the echo SNR = 15 dB under typical scanning conditions, and that all significant tissue motion is in the scan plane, we averaged signal power over 2 s at 8 fps and 3 mm^2^ of tissue to indicate the steady-state perfusion with acceptable precision [20], [25]. When necessary, the precision of power estimates is improved by averaging more echo frames from each Doppler frame.

Once the IQ signals have been converted into RF echo data according to manufacturer instructions [55], the 16 echo frames in each 2 s interval are spatially registered to the first echo frame to minimize decorrelation of tissue echoes from tissue motion occurring between Doppler frames. For this task, a rigid spatial registration method provides sub-sample RF echo-signal registration accuracy with micron-scale precision [20]. In Fig. 4(b), we show an example of axial and lateral displacements estimated before and after spatial registration for one mouse experiment. The tissue movement in this example is synchronous with respiration, where the scan plane periodically translates 0.010 mm in a direction lateral to the beam axis and along the muscle fibers while trending downward along the beam axis and to the left toward the foot.

Finally, spatially registered data are passed through a principal component analysis (PCA) clutter filter before estimating signal power in the region [20] and generating PD-US images [19]. We found that when the tissue is motionless over the 2 s interval, eliminating the first singular value in the PCA filter output minimizes tissue clutter power [20], [25]. Hence, blood cell motion is the principal source of decorrelation for echo signals that have passed through the PCA filter. The post-filtered echo-signal power is computed for each hindlimb and normalized by its pre-ligation baseline value. The mean and standard errors are computed for the sample size, and the results are converted to decibels and plotted in Fig. 6.

We obtained results with and without spatial registration applied before PCA clutter filtering. In situations where tissues translate in the scan plane more than 10–20 μm, spatial registration improves the separation of the signal subspaces associated with strong slow-moving tissue echoes and weak slow-moving blood-cell echoes. Effective clutter filtering is essential for PD-US estimates to reproducibly represent blood perfusion without contrast enhancement. Although PD-US estimates do not provide quantitative estimates in units of flow per mass of tissue, they do provide signal-power estimates that reproducibly follow perfusion changes occurring over the four-week study period [25].

### G. Parameters Characterizing the Ischemic Response

The results section displays the time series of hindlimb muscle perfusion changes measured during the 28-day post-ligation measurement schedule of Fig. 2. This section describes three parameters estimated from those results. The parameters are selected to characterize the average response of the hindlimb vasculature to right femoral ligation in healthy and diabetic animals. They are the spike in perfusion observed in all animals 20 min after ligation *P*_max_, the minimum perfusion index occurring 1–2 days post-ligation relative to baseline *P*_min_, and the duration of the ischemic period as measured by the time required for perfusion to return to the baseline value *T*.

**Fig. 5.**
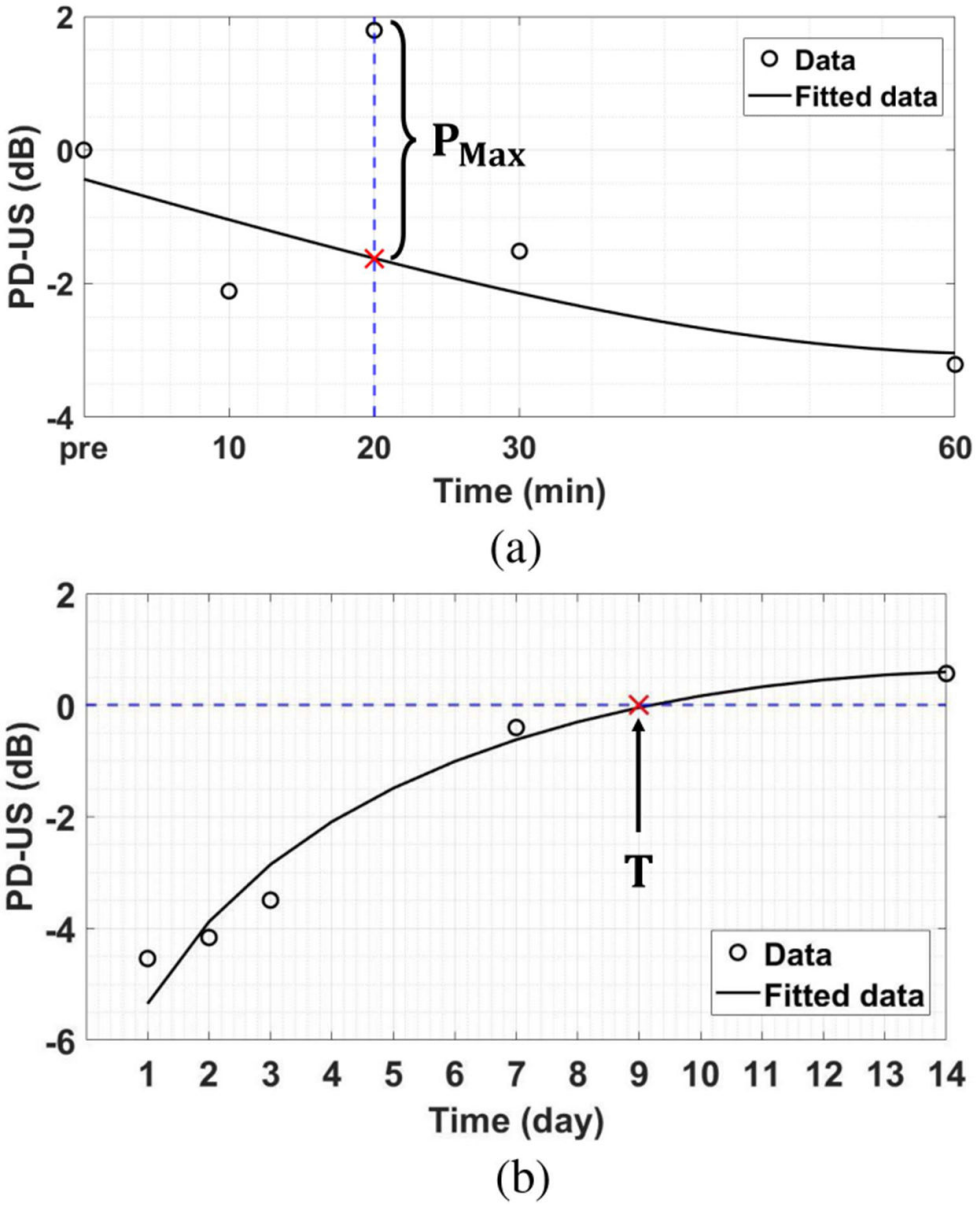
(a) Points are perfusion measurements made before (pre) and at four times after right femoral ligation. The solid line is a polynomial fit to these four points. *P*_max_ is the difference (in dB) between measured and fit values at 20 min. The 3.5 dB shift indicates a two-fold increase in perfusion-related signal power at 20 min relative to the trend between 10–60 min. (b) Example of a recovery time measurement *T* from the polynomial fit of perfusion data, where 1 ≤ *t* ≤ 14 days. In this example *T* = 9 days. The points in both plots are from the data in Fig. 6.

Perfusion in the ischemic hindlimb, as estimated by PD-US values, declines progressively during the first 24–48 hours. However, at the 20-min time point, perfusion briefly doubles. To estimate the 20-min perfusion burst *P*_max_, we fit PD-US measurements made prior to ligation and at 10, 30, and 60 min post-ligation to a second-order polynomial. *P*_max_ is given by the difference (in dB) between measured and curve-fit values, as illustrated in Fig. 5(a). The error bar for *P*_max_ estimates is given by the standard deviation of PD-US measurements at 20 min.

Similarly, to estimate the time at which perfusion in each mouse returns to its pre-ligation baseline value, we fit PD-US estimates of perfusion measured 1, 2, 3, 7, and 14 days post-ligation to a second-order polynomial. An example is shown in Fig. 5(b). Since PD-US points are measured values relative to the average baseline value, recovery time *T* is estimated at the point the fit line crosses the 0-dB perfusion axis. Finally, *P*_min_ is the minimum PD-US value measured during the 28-day experiment.

### H. Statistical Analyses

**Fig. 6.**
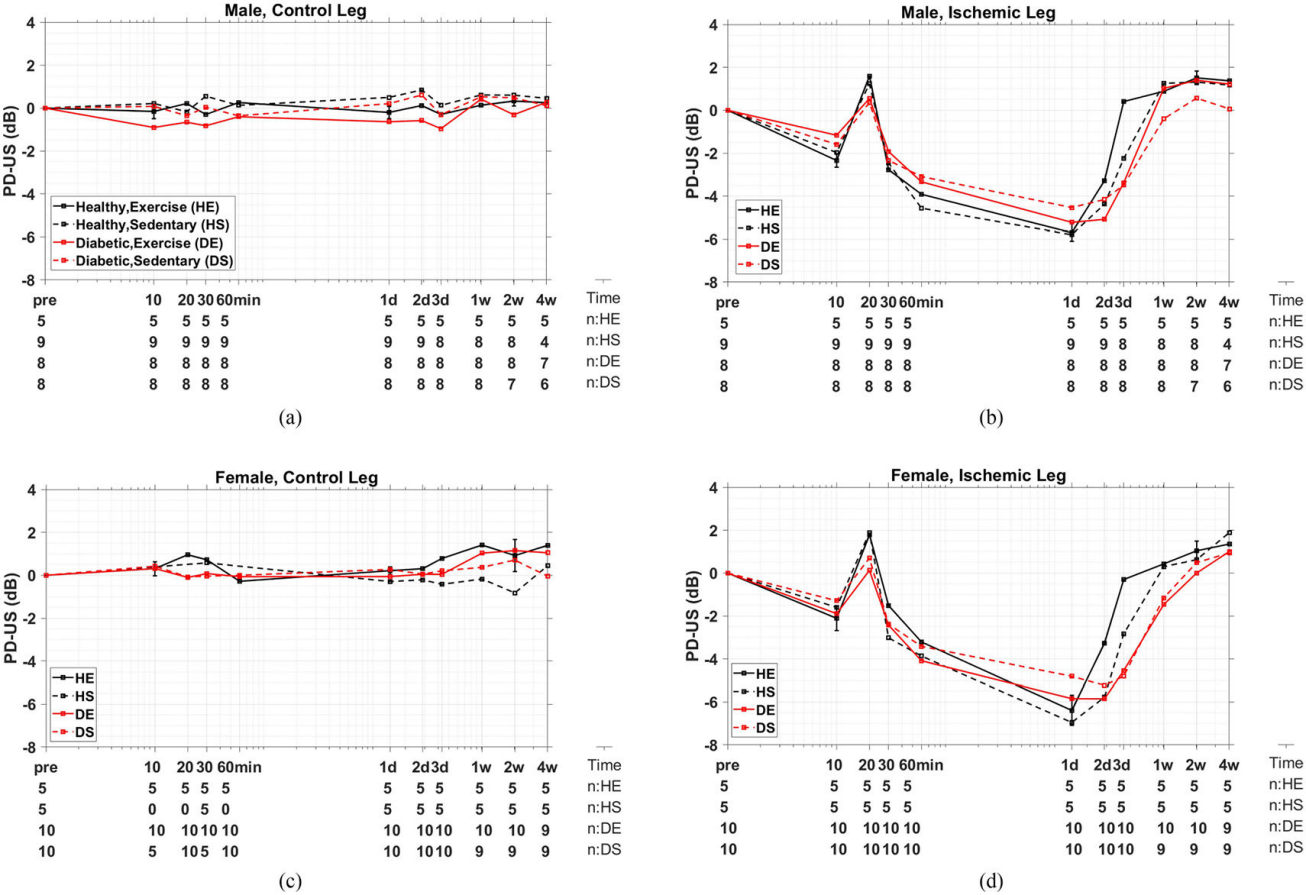
Power Doppler ultrasound (PD-US) measurements for male (a), (b) and female (c), (d) mice as a function of time are plotted on log-log scales. Measurements are made on the non-ligated (control) left leg (a), (c) and the ligated (ischemic) right leg (b), (d). Black lines indicate perfusion changes relative to the pre-ligation baseline in healthy (non-diabetic) mice while red lines indicate that for diabetic mice. Solid lines indicate results for exercised mice and dashed lines for sedentary mice; e.g., DE labels the curve for the diabetic exercised group while HS labels the healthy sedentary group. The time axis extends from pre-ligation baseline measurements (pre) through four weeks post-ligation (4 w) as indicated by the schedule in Fig. 2. Below the time axis are sample sizes (*n*) over the 4-week experiment for the four mouse groups. Animal numbers vary because of attrition and unavailable data. Error bars at 10 min, 1 d, and 2 w for the HE mouse group indicate ±1 se.

The experimental variables influencing measurement results are listed in Table I, except for the time-varying sample sizes *n* listed in Fig. 6. All statistical analyses were performed in Matlab.

Each PD-US measurement is the clutter-filtered, baseline-normalized, mean echo-signal power describing perfusion during a 12 s acquisition (average of 6 sequential 2 s estimates). The time points plotted in Fig. 6 further average PD-US measurements over sample size *n*, the number of mice for each group as listed below the plots. Representative error bars denote ±1 standard error (se).

Mice in some diabetic groups did not all survive the four-week study. To restore the falling sample sizes, we added a second study with mice from the original cohort that were treated in the same way but studied months later (see Table I). Because perfusion may be influenced by mouse weight and age [39], we tested the two data sets for statistical differences using non-parametric analysis of covariance (ANCOVA) [36]. ANCOVA evaluates whether the mean PD-US estimates for these two groups are equivalent, while statistically controlling for mouse age and weight as continuous covariates. Finding that we could accept the null hypothesis of equal means at the *p* = 0.05 significance level, the two groups were combined to increase the sample sizes for statistical testing as indicated in the right-most column of Table I.

We evaluated the need to apply spatial registration by processing the perfusion profiles appearing in Fig. 6 in two identical ways with one exception: in one set of profiles, we applied spatial registration before PCA clutter filtering and, in the other set, filtering was applied without first registering the echo frames. If no significant differences between curves from comparable groups are found, we may assume that any tissue motion occurring as echo frames are acquired is negligible. A Box-Cox transformation ((*x*^*λ*^ − 1)*λ* for data point *x* and 0.1 < *λ* < 1.1) [37] was applied to mean PD-US measurements for all points in a given perfusion profile to improve the normality of data and assume the samples at each time have equal variances. We then applied a parametric two-sided t-test and a non-parametric Kruskal-Wallis test [38] to evaluate differences between curves found with and without registering the echo frames. We further applied the Wilcoxon Rank Sum Test [38] to evaluate differences between median values. We were unable to reject the null hypothesis at the *p* = 0.05 significance level for all three tests. Hence, when considering the entire perfusion profile in the analysis, spatial registration before clutter filtering did not significantly modify the PD-US-based perfusion profiles.

We also evaluated the mean differences between registered and unregistered data at individual time points using the Wilcoxon rank sum test applied without the Box-Cox transformation. Only five of the 88 point-wise comparisons suggested spatial registration before clutter filtering resulted in a significantly different mean value compared with unregistered data. Outliers often occurred near the perfusion profile minima, near days 1 and 2. So, we used spatially registered PD-US profiles to compare ischemic parameters (described in Section II-G, with results in Figs. 9-11) because just a portion of the perfusion profile determined those parameter estimates.

In the results below, the null hypothesis was tested only between two groups that differed by one biological variable. For example, we tested for differences between healthy and diabetic mice within the sedentary female groups, but we did not test for differences between sedentary diabetic males and exercised healthy females.

## III. Results

### A. Temporal Perfusion Profiles

Fig. 6 summarizes the PD-US measurements describing muscle perfusion over the four-week study relative to pre-ligation baseline values (pre). The abscissa marks the time after the right femoral ligation. Spatial registration was not applied prior to PCA clutter filtering when generating these data. The measurements are for male (a), (b) and female (c), (d) mice, where control measurements on the intact left hindlimb (a), (c) may be compared with measurements made on the ischemic right hindlimb (b), (d) following femoral ligation. The four curves on each graph describe results for the healthy exercised (HE) and sedentary (HS) mouse groups and the diabetic exercised (DE) and sedentary (DS) groups. Below the time axes are listed the sample sizes for each group as they varied during the study.

### B. Spatial Registration and the PCA Clutter Filter Threshold

We found that rigidly fixing the position of the ultrasound probe and gently binding the hindlimbs with Velcro straps was effective at minimizing clutter (tissue) motion in these studies. That is, we found no statistically significant differences between the results in Fig. 6 when echo frames are spatially registered or not. This finding is in contrast to an invasive vascular study [25], where in-plane tissue motion greater than 0.01 mm was common and found to modify the PD-US signal unless the echo frames were spatially registered.

**Fig. 7.**
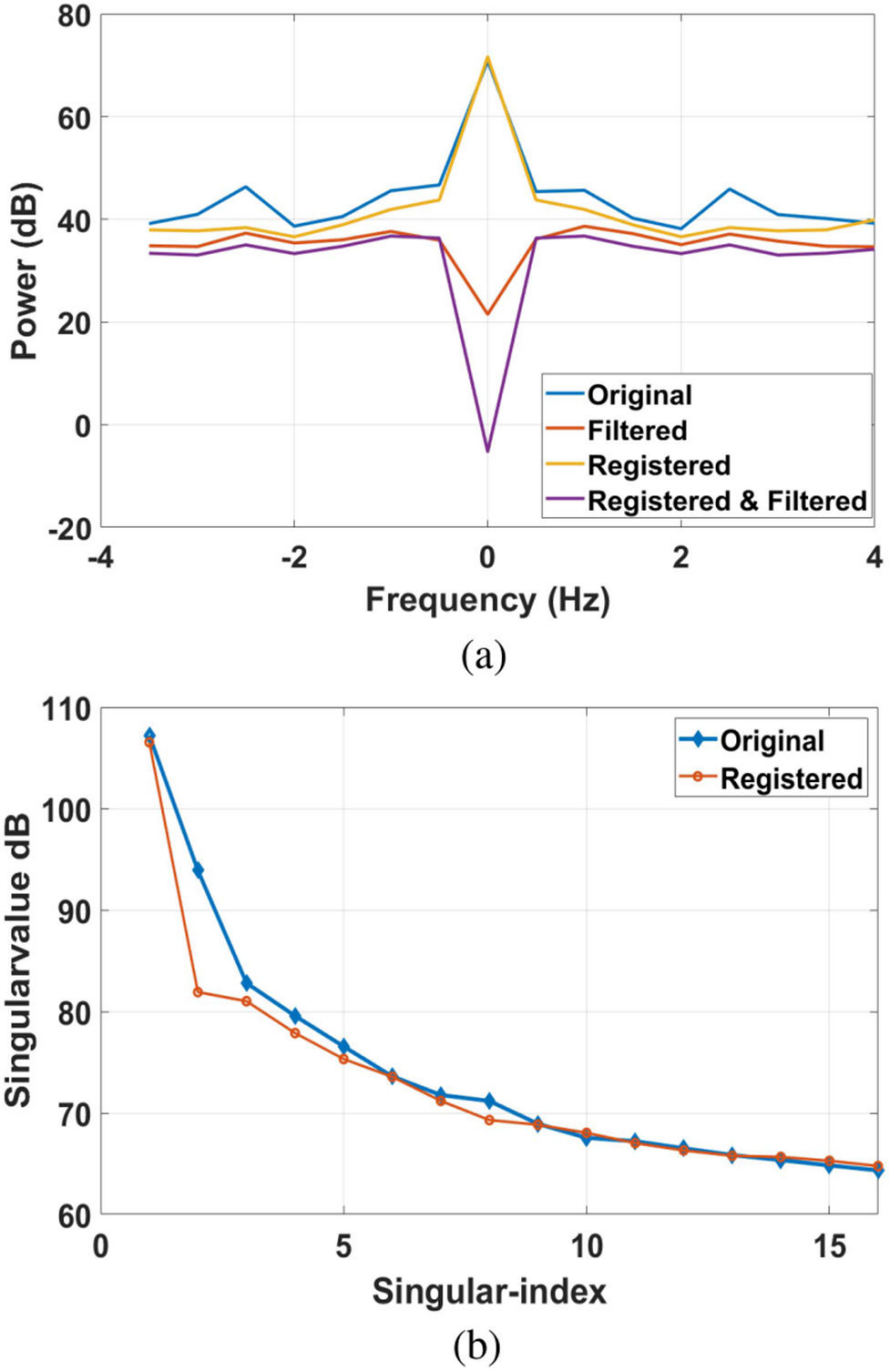
(a) Doppler spectra are displayed at different levels of processing. “Original” indicates the power spectral density for echo data as it was recorded. “Registered” labels the spectrum obtained after spatially registering the 16 echo frames in a 2 s acquisition. “Filtered” and “Registered & Filtered” are spectra obtained after applying a PCA clutter filter without and with spatial registration, respectively. (b) Singular spectra are shown from SVD processing of the same data shown in (a) with and without spatial registration.

**Fig. 8.**
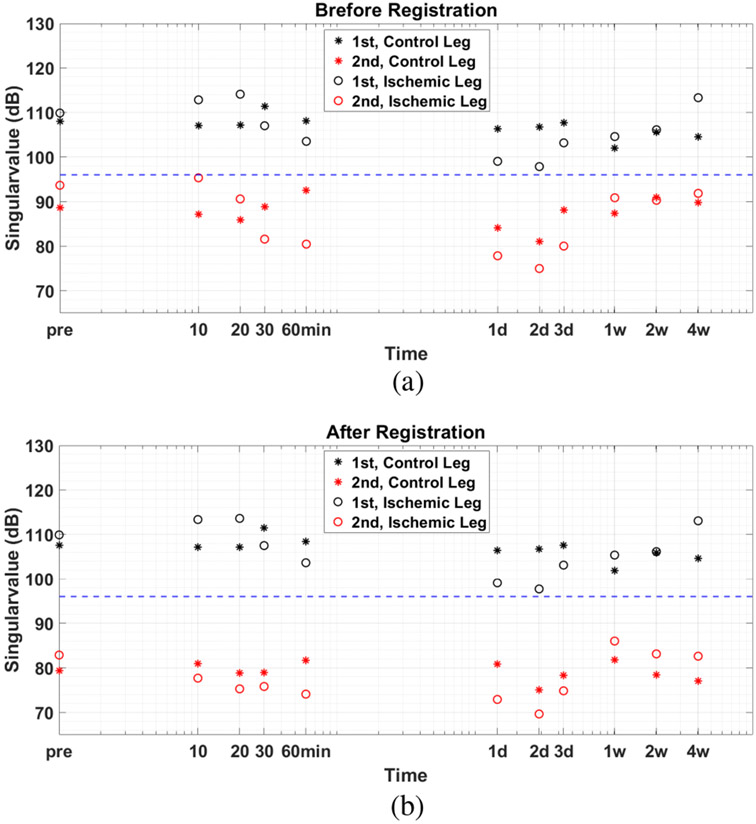
First two singular values from singular spectra obtained at each muscle measurement time are plotted for the ischemic (right) and control (left) hindlimbs. Comparing values before spatial registration of the echo frames (a) with those after registration (b) shows that spatial registration increases the difference between the first and second singular values. Greater separation generates less overlap between the clutter and blood subspaces, thus increasing the reliability of clutter filtering.

Figs. 7 and 8 help explain why spatial registration had minimal influence on PD-US estimates in this study. Fig. 7 displays one representative Doppler spectra at various stages of processing. The pre-filtered “original” and “registered” spectra are both symmetric about zero frequency, which is a characteristic of diffuse blood-cell-scatterer motion. Peaks in the original spectrum at ±2.5 Hz that are associated with respiration are effectively minimized after spatial registration. Nevertheless, the PCA “filtered” and “registered & filtered” spectra are similar to each other whether or not the echo frames are first spatially registered. In both cases, the PCA filter threshold is set to pass all singular values except the first. PD-US estimates are equal to the area under the filtered Doppler spectrum.

Fig. 7(b) displays the singular-value spectra analogous to the Fourier spectra of Fig. 7(a). The “original” singular spectrum is generated before spatial registration is applied and the “registered” spectrum is after registration. The greatest influence of registration on PD-US estimates is to increase the separation between the first two singular values, which was selected as the boundary between the clutter-echo and blood-echo subspaces.

The first two singular values are plotted for all data recorded during the four-week study in Fig. 8. We find that spatial registration increases the difference between the first two singular values, which signifies a reduction in the overlap between the clutter and blood subspaces. Nevertheless, the same filter threshold (dashed lines) clearly separates the first and second singular values at every point in time whether we use registration or not. Coherent tissue motion less than 0.01 mm does not change the PCA filter threshold between the clutter and blood subspaces. With greater tissue motion, one threshold will no longer separate adjacent singular values unless the echo signals are first spatially registered. We note that the correlation coefficient among the pre-filtered echo frames remained above 0.8 for unregistered frames and above 0.9 for registered frames, further indicating that little tissue motion occurred between frames [20].

Fig. 7(a) shows that the filtered power-spectral density at zero frequency is substantially different with and without registration. However, that difference, which is roughly the same for all measurement times during the 4-wk study, is removed by normalizing PD-US estimates by the pre-ligation baseline value.

The next section examines the sensitivity of each perfusion profile to diabetic changes, including the influence of sex and fitness level.

### C. Perfusion Profiles for Healthy and Diabetic Hindlimb Muscles

The perfusion profiles for the ligated right hindlimb (Fig. 6(b) and (d)) initially decrease until, at 20 min post-ligation, there is a sudden 2–3 dB burst of perfusion for all mouse groups. Subsequently, perfusion continued to decline between 30 min and 1–2 days to a minimum value of −5 dB to −7 dB (20–32% of baseline). Perfusion was found to recover to baseline values in 3–14 days, with a rate that is correlated with group properties.

Fig. 6(a) and (c) show no significant changes in muscle perfusion among mouse groups for the left hindlimb that serves as a control measurement. Perfusion remained constant within ±1 dB of baseline, showing that right femoral ligation did not generate systemic effects appearing as changes in left peripheral muscle perfusion.

No significant differences in baseline perfusion measurements were found among the eight groups (data not shown), except the baseline PD-US estimates in females were consistently 2–3 dB greater than estimates in males.

### D. Minimum Perfusion P_min_

**Fig. 9.**
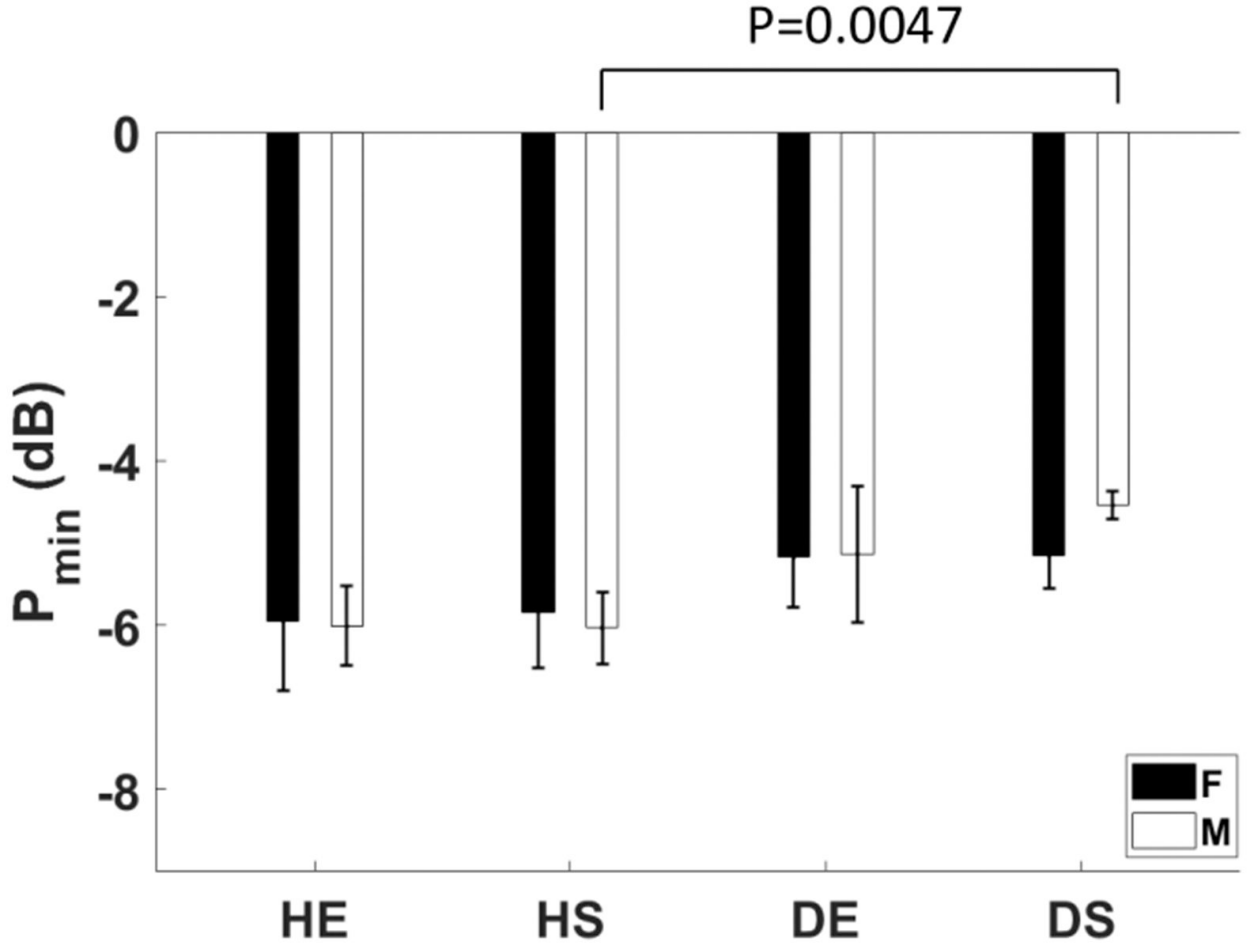
Average reduction in perfusion relative to baseline within the right hindlimb muscles, *P*_min_, is shown for the eight mouse groups. Significant differences are indicated by *p* values between compared groups. Error bars indicate ±1 se.

Fig. 9 shows that all mouse groups experienced reduced perfusion following ligation to a value between one-fifth and one-third baseline. Although there is a nominally greater reduction in healthy animals compared to diabetic animals, the only statistically significant difference was between the healthy and diabetic sedentary males.

### E. Recovery Time T

**Fig. 10.**
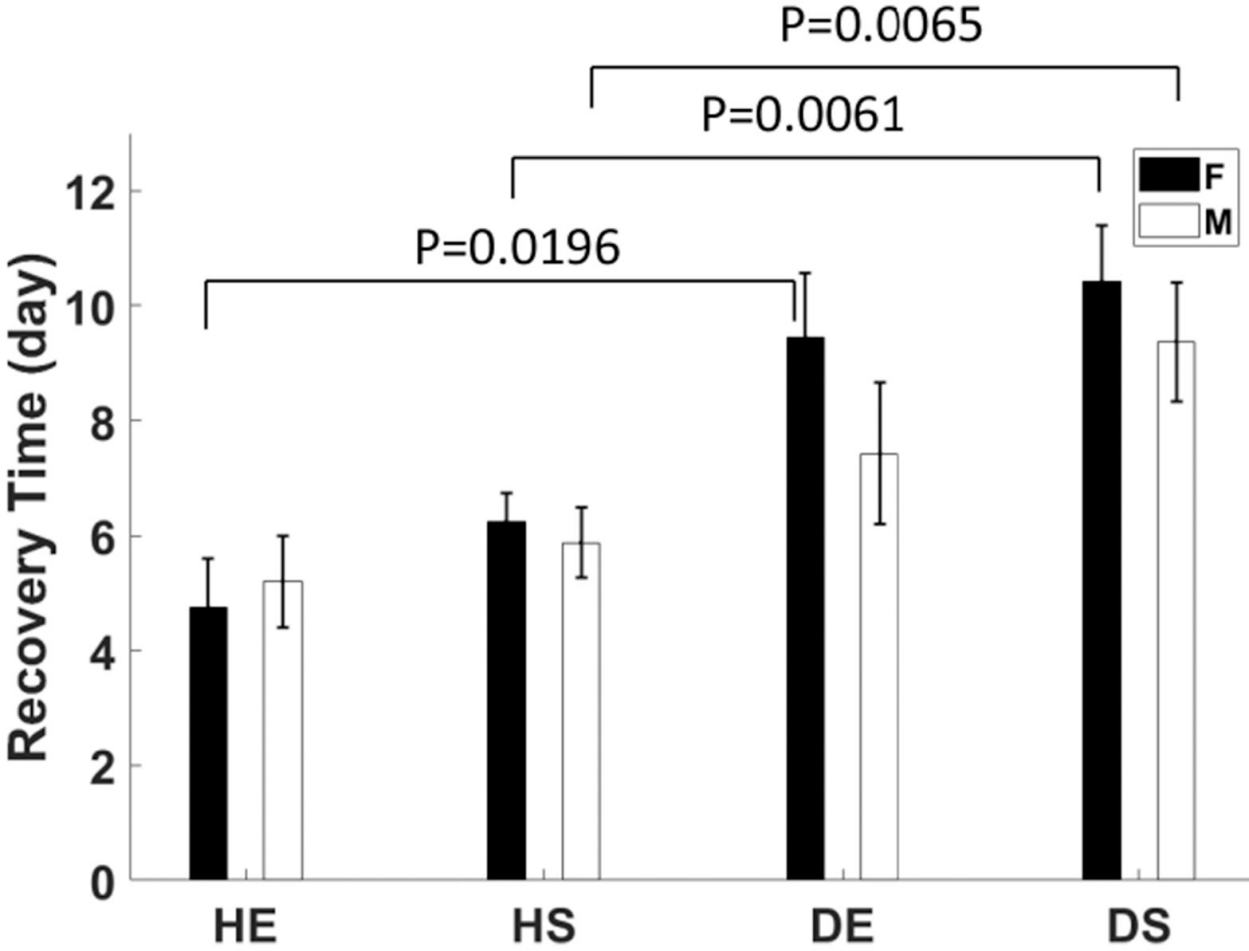
Time following ligation when perfusion returned to baseline values *T* [days] is shown for the eight mouse groups. Significant differences (*p* < 0.05) were found between healthy and diabetic females and diabetic males. Error bars indicate ±1 se.

Fig. 10 shows that muscle perfusion recovered more quickly in healthy female mice compared to diabetic female mice, whether they exercised or not. The trend for males is similar. It appears that diabetic females require more time to recover baseline perfusion compared with diabetic males and that diabetic animals that exercised recovered more quickly than sedentary diabetic animals, but these trends were not statistically significant.

### F. P_max_, FMD, and RH

**Fig. 11.**
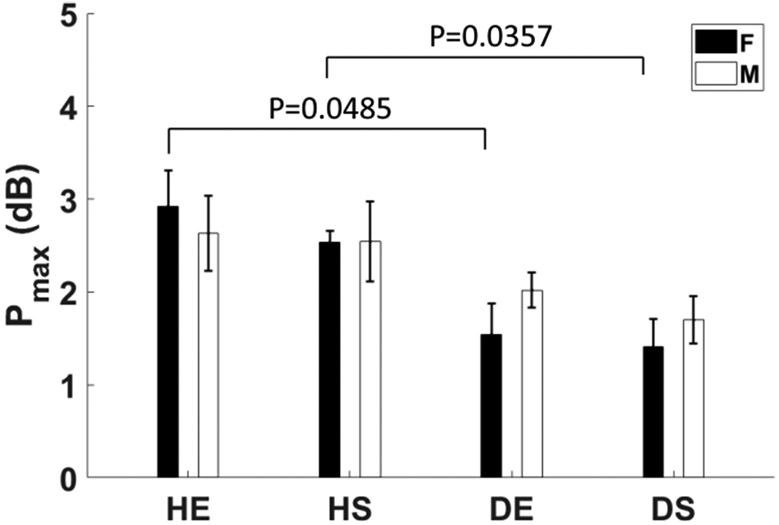
Magnitude of the spike in muscle perfusion appearing 20 min post-ligation *P*_max_ in Fig. 6 is shown for the eight mouse groups. Error bars indicate ±1 se.

A prominent feature of perfusion profiles in the ischemic hindlimbs of Fig. 6(b) and (d) is a 2–3 dB spike in muscle perfusion appearing 20 min after ligation. The height of perfusion spikes for all eight groups are summarized in Fig. 11 as *P*_max_ estimates. Significant differences between paired healthy and diabetic groups are indicated.

**Fig. 12.**
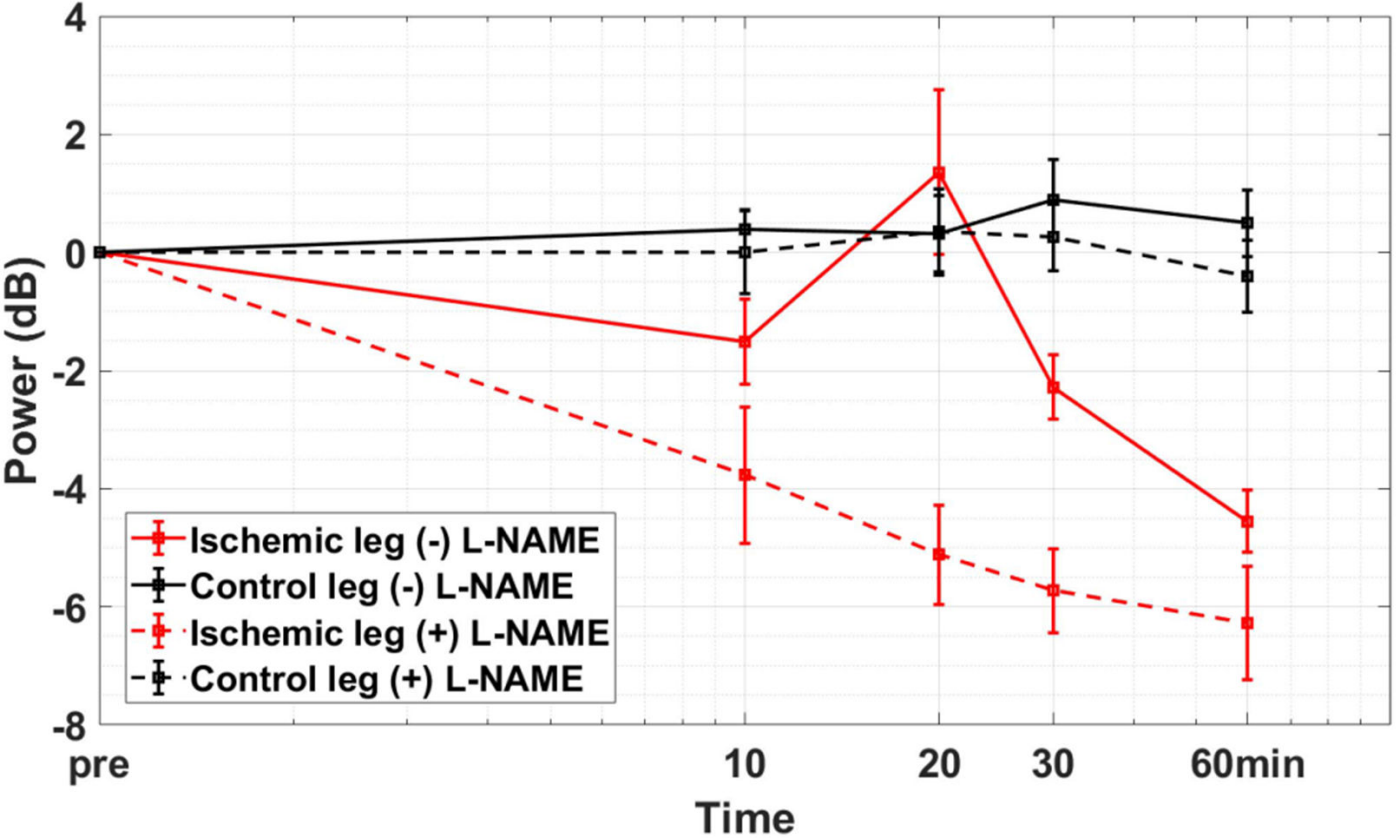
PD-US measurements for the ischemic hindlimb (red) and control hindlimb (black) up to 60 min post ligation for healthy sedentary male mice. The dashed lines describe perfusion in mice receiving an i.p. injection of NOS inhibitor L-NAME 60 min before ligation (*N* = 5). Solid lines describe perfusion in mice without L-NAME injections (*N* = 4). Error bars indicate ±1 se.

It is possible that the brief surge in perfusion is related to changes in resistance artery/arteriole tone from nitric oxide (NO) released in collateral vessels adjusting to the sudden ischemia following femoral ligation. This assumption was confirmed in a side study with results summarized in Fig. 12. We repeated mouse perfusion measurements during the first 60 min after ligation, which are shown by solid lines representing the average PD-US estimates in the ligated and control hindlimb of 4 mice. We then injected the nitric oxide synthase (NOS)-inhibitor L-NAME into 5 additional mice 60 min before ligation and repeated the measurements. We find the 20-min perfusion peak is highly correlated with endothelium-dependent NO activity given than L-NAME completely eliminated the 20-min peak without significantly altering perfusion in the control hindlimb. Consequently, it is reasonable to assume that the lower peak perfusion seen for diabetic mice in Fig. 11 results from NO-related endothelial dysfunction.

**Fig. 13.**
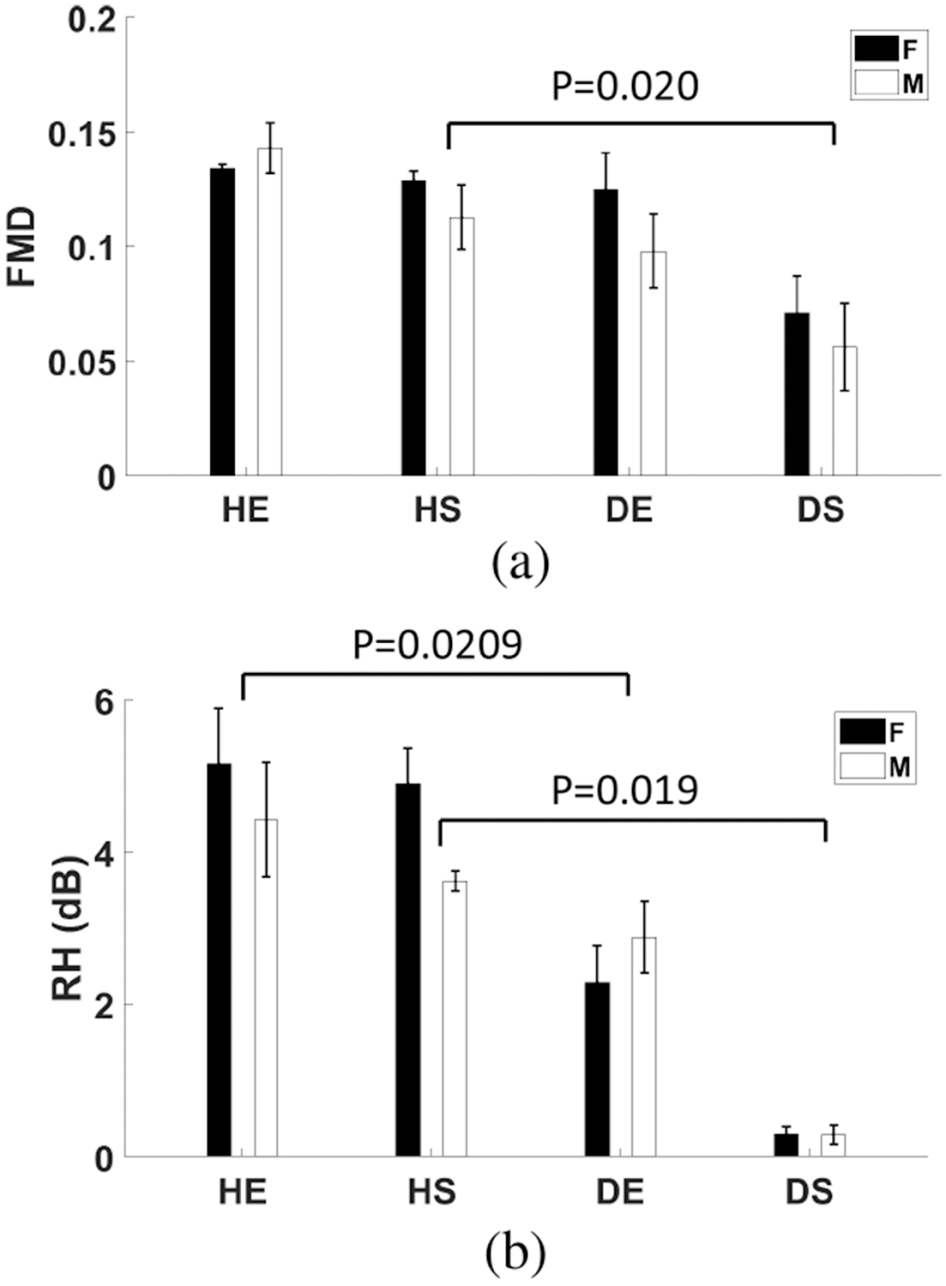
(a) Flow-mediated dilation (FMD) of the femoral artery applied to the control (left) hindlimb before right femoral ligation. (b) Reactive hyperemia (RH) index estimated for the same groups measured at the same time as FMD describe the peak in excess muscle perfusion following 5 min pressure cuff occlusion. Error bars indicate ±1 sd.

Next, we tested to see if the loss in endothelial function was concentrated in conduit or resistance vessels by measuring both FMD and RH index; see Fig. 13. Both measurements are made prior to femoral ligation using a 5-min pressure-cuff occlusion (Section II-D). The results of Fig. 13 show a reduction in FMD in diabetic sedentary mice compared with healthy sedentary mice. The findings suggest that exercise provides some protection against endothelial dysfunction in the conduit arteries of diabetic mice.

Fig. 13(b) further shows there is a reduction in the RH index for all diabetic groups, indicating the endothelial dysfunction extends to resistance vessels.

The data in Figs. 11 and 13 are consistent regarding diabetic changes in endothelial function. However, FMD and RH are more sensitive indicators than *P*_max_. We combined the male and female groups for the measurements reported in Fig. 13 to increase the sample size to 10.

### G. Changes in Body Weight

Belcik et al. [42] found that insulin resistance, which is generally associated with increased body weight, is correlated with reduced microvascular blood flow and capillary blood volume in skeletal muscle. Their finding prompted our investigation into the complicating influence of body weight when comparing the perfusion profiles of healthy and diabetic mice.

**Fig. 14.**
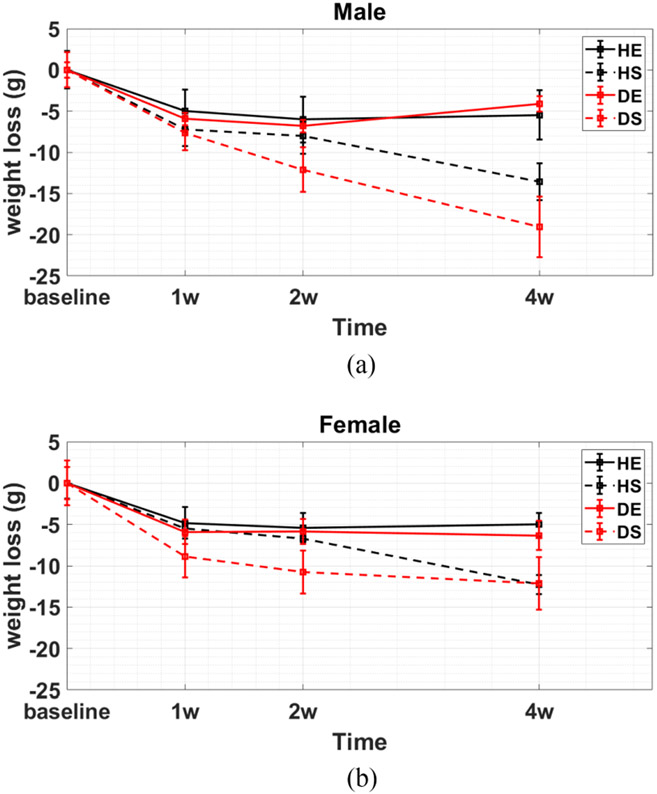
Change in mean body weight for each of the eight mouse groups during the four-week study. While all mice lost at least 4 g during the first week after ligation, the weight of animals that exercised stabilized while that for sedentary mice declined.

Table I shows that the diabetic groups were significantly heavier than healthy mice at the time of the baseline measurements. Fig. 14 further shows that all mice lost 4–8 g during the week following right femoral ligation. While body weight continued to fall over the four-week trial in all sedentary animals, it stabilized in animals that maintained an exercise schedule. Exercise is the variable most correlated with time-dependent weight loss for the eight groups, and yet exercise had no significant effect on perfusion as reported in Figs. 9-11. Consequently, we conclude that variations in mouse body weight had little effect on the relative perfusion profiles.

## IV. Discussion

The results presented show that PD-US estimates are sensitive to the diabetes-related changes in perfusion measured during ischemic challenges. We found that all mice in the study recovered their baseline levels of muscle perfusion within four weeks of femoral ligation. However, the perfusion recovery time *T* increased an average of 47% in diabetic males and 74% in diabetic females compared with their non-diabetic counterparts. Other investigators [44], [45] observed incomplete recovery after 14 days when monitoring perfusion at the surface of the foot and calf muscles in diabetic mouse models compared with non-diabetic controls. Although the specific ischemic response may be expected to vary with details of each experimental approach, the findings of diminished and delayed perfusion recovery are consistent with the vascular endothelial damage expected during persistent hyperglycemia in insulin-resistant mice [46].

It was reported [41] that exercise training improves the overall endothelial function in a group of human subjects with no clinical evidence of vascular disease. We found exercise preserved endothelial function in diabetic mice (Fig. 13). Otherwise, our data show fitness has a minimal effect on the perfusion profile describing ischemic recovery.

The body weight in exercised animals stabilized within a week following surgery, whereas sedentary animals continued to lose weight over all four weeks, as seen in Fig. 14. This finding correlated with our observation that healthy animals at any fitness level tended to be more active than diabetic animals after surgery. Greater activity correlated with stable body weight and deeper ischemia (lower values of *P*_min_ in Fig. 9).

The most consistent parametric differences observed were between sedentary healthy and sedentary diabetic mice for both the male and female groups. The sedentary diabetic animals were older, as seen in Table I, and age is a complicating factor in perfusion recovery along with diabetes. For example, Farber et al. [51] found that healthy older mice had fewer and smaller-diameter native collateral vessels, which increased the severity of the ischemic injury following ligation and slowed perfusion recovery. Westvik et al. [52] also found delayed ischemic hindlimb recovery in older healthy mice compared to younger healthy mice. Although older mice exhibited higher capillary density, suggesting a vigorous angiogenic response to ischemia, perfusion recovery was nevertheless attenuated because of a significantly impaired arteriogenic response.

The long-term loss of endothelium-dependent vasodilation in conduit and resistance arteries promotes the development of PAD, which increases a patient’s risk of developing coronary artery and cerebrovascular diseases [40], [50]. There is no evidence that mice developed PAD during this experiment, but the diabetes-related endothelial dysfunction that was apparent in mice is prominent in patients developing PAD. Consequently, efforts to resolve intermittent claudication in humans are shifting to the improvement of endothelial function [48], especially in resistance vessels most effected by metabolic disease [9].

Peripheral arterial tonometry (RH-PAT) is a clinical technique for assessing endothelial function in resistance vessels [9], [43], [49]. RH-PAT devices measure arterial pulsatile volume changes non-invasively at the fingertip or forearm during reactive hyperemia following short-duration ischemic periods [47]. PD-US imaging is a site-specific alternative to RH-PAT, where the ultrasonic echo-signal power originating primarily from the motion of red blood cells is spatially mapped.

The intra-subject coefficient of variation (CV) for RH-PAT measurements in young human males was reported to be in the range of 15–22% [43]. In comparison, the CV for PD-US-based RH measurements from the healthy male mouse data of Fig. 13(b) is 8–29%, increasing to 16–46% in diabetic males. PD-US measurement variability is notably influenced by the reproducibility of selecting a region of interest in muscle, day-to-day activity-related perfusion changes, and our ability to minimize tissue motion during echo acquisition to maximize the effectiveness of PCA clutter filtering.

We have not found any literature reports of the 20-min perfusion peak seen in the data of Fig. 6 and quantified in Fig. 11. Fig. 12 clearly shows that the peak is related to NO-mediated dilation. The height of the peak is related to endothelial function but that measurement is less sensitive than FMD and RH estimates implemented via PD-US measurements.

PD-US assessments are a viable alternative to dynamic contrast-enhanced ultrasonic (DCE-US) imaging [53], [54] when the clinical objective is to frequently monitor regional changes in muscle perfusion over time. Applications include evaluating PAD patients for atherosclerosis progression and treatment responses. DCE-US remains a critical tool for quantitative measurements of perfusion or for neurovascular applications requiring visualization of blood flow within the microvasculature.

## V. Conclusion

PD-US without contrast enhancement provides reproducible measurements of changes in peripheral muscle perfusion over time. The principal challenges to measurement reproducibility over weeks and months are a consistent selection of the muscle region for analysis and the elimination of tissue-clutter motion between echo frames used to estimate power-Doppler signals. PD-US-based estimates of RH index are made following 5-min ischemic periods induced by a pressure cuff. Combining PD-US estimates of RH with standard FMD measurements, a more comprehensive assessment of endothelial function emerges. These measurements can help physicians monitor a patient’s risk of developing systematic atherosclerosis, which is a measure of overall cardiovascular health.

## Figures and Tables

**TABLE I:** Summary of Mouse-Group Information.

Group	Date of Experiment	Age (months)	Ligation type	Sex	Avg. Weight (g)	*N*′	*N*
Healthy, Sedentary (HS)	8.25.2020	10	FA	F	NA	5	F = 5
				M	NA	4	M = 8
	6.21.2023	9	FA&V	M	34.1	4	
Diabetic, Sedentary (DS)	10.5.2020	12	FA&V	F	56.82	4	F = 9
				M	63.26	2	
	11.5.2021	25	FA&V	F	54.6	5	M = 6
				M	55.86	4	
Diabetic, Exercise (DE)	7.2.2021	7	FA&V	F	44.08	4	F = 9
				M	46.64	5	
	11.19.2021	11	FA&V	F	48.68	5	M = 7
				M	53.52	2	
Healthy,Exercise (HE)	2.18.2022	14	FA&V	F	32.76	5	F = 5
				M	45.78	5	M = 5

The sources of variability in this study are represented as columns. Values in columns listed as Date of Experiment, Age, Avg. Weight are baseline values at *t* = 0. *N*′ is the number of mice that survived the 4-week trial out of 5 (in one case 10). *N* indicates mouse numbers for Males and Females after combining data from different experiments within one Group. FA — femoral artery, FA&V = femoral artery and vein.
